# Genome, secretome and glucose transport highlight unique features of the protein production host *Pichia pastoris*

**DOI:** 10.1186/1475-2859-8-29

**Published:** 2009-06-02

**Authors:** Diethard Mattanovich, Alexandra Graf, Johannes Stadlmann, Martin Dragosits, Andreas Redl, Michael Maurer, Martin Kleinheinz, Michael Sauer, Friedrich Altmann, Brigitte Gasser

**Affiliations:** 1 Department of Biotechnology, University of Natural Resources and Applied Life Sciences, Vienna, Austria; 2 School of Bioengineering, University of Applied Sciences FH-Campus Wien, Vienna, Austria; 3 Department of Chemistry, University of Natural Resources and Applied Life Sciences, Vienna, Austria

## Abstract

**Background:**

*Pichia pastoris *is widely used as a production platform for heterologous proteins and model organism for organelle proliferation. Without a published genome sequence available, strain and process development relied mainly on analogies to other, well studied yeasts like *Saccharomyces cerevisiae*.

**Results:**

To investigate specific features of growth and protein secretion, we have sequenced the 9.4 Mb genome of the type strain DSMZ 70382 and analyzed the secretome and the sugar transporters. The computationally predicted secretome consists of 88 ORFs. When grown on glucose, only 20 proteins were actually secreted at detectable levels. These data highlight one major feature of *P. pastoris*, namely the low contamination of heterologous proteins with host cell protein, when applying glucose based expression systems. Putative sugar transporters were identified and compared to those of related yeast species. The genome comprises 2 homologs to *S. cerevisiae *low affinity transporters and 2 to high affinity transporters of other Crabtree negative yeasts. Contrary to other yeasts, *P. pastoris *possesses 4 H^+^/glycerol transporters.

**Conclusion:**

This work highlights significant advantages of using the *P. pastoris *system with glucose based expression and fermentation strategies. As only few proteins and no proteases are actually secreted on glucose, it becomes evident that cell lysis is the relevant cause of proteolytic degradation of secreted proteins. The endowment with hexose transporters, dominantly of the high affinity type, limits glucose uptake rates and thus overflow metabolism as observed in *S. cerevisiae*. The presence of 4 genes for glycerol transporters explains the high specific growth rates on this substrate and underlines the suitability of a glycerol/glucose based fermentation strategy. Furthermore, we present an open access web based genome browser .

## Background

Yeasts have attracted renewed interest in the last few decades as production hosts for biopharmaceutical proteins as well as for bulk chemicals. The methylotrophic yeast *Pichia pastoris *(Guillermond) Phaff (1956) is well reputed for efficient secretion of heterologous proteins [1], and has come into focus for metabolic engineering applications recently. Especially reengineering of the N-glycosylation pathway has enabled the production of heterologous proteins with human-like N-glycan structures [2-4]. While protein production is the major application of *P. pastoris*, production of metabolites has come into research focus recently too [5,6]. Apart from these biotechnological applications, it is widely used as a model for peroxisome [7] and secretory organelle research [8]. *P. pastoris *has recently been reclassified into a new genus, *Komagataella *[9], and split into three species, *K. pastoris*, *K. phaffii*, and *K. pseudopastoris *[10]. Strains used for biotechnological applications belong to two proposed species, *K. pastoris *and *K. phaffii*. The strains GS115 and X-33 are *K. phaffii*, while the SMD series of protease deficient strains (most popular SMD1168) is classified into the type species, *K. pastoris*. Apart from these strains which have been made available by Invitrogen, research labs and industry use different other strains belonging to either of these two species, and no trend towards a superior expression level of one of the two species has been observed. In order to provide a common information basis across the different strains, we have performed this work with the type strain (DSMZ 70382) of the type species *K. pastoris*, which is the reference strain for all the available *P. pastoris *strains. In coherence with the published literature, we name all strains *P. pastoris*, which thus stands for the entire genus *Komagataella*. As other strains, DSMZ 70382 was isolated from tree exudate, in this case from the chestnut tree.

The majority of *P. pastoris *processes described so far utilize methanol as substrate and inducer for heterologous protein production. While tight gene regulation and high product titers can be achieved with this strategy, the disadvantages as large scale use of a flammable substrate, high heat production and oxygen consumption, and significant cell lysis have been reported. Apart from technological challenges in large scale fermentation, this leads to significant contamination of culture supernatants with intracellular proteins including proteases [11]. *P. pastoris *has been described to secrete some heterologous proteins like human serum albumin [12] or as recently reported glycoengineered antibodies [13] in the g L^-1 ^range, while naturally secreted proteins account only for low amounts [14], which supports the easy production of highly pure proteins. However, several secreted *P. pastoris *proteins are observed as contaminants in culture supernatants, requiring elaborate product purification and analytical effort. A detailed characterization of the secretome would significantly improve production and quality control of biopharmaceuticals produced with this expression system. The secretomes of few yeasts and filamentous fungi have been analyzed experimentally. Computational analyses of yeast genomes predicted approximately 200 potentially secreted proteins [15,16]. Secretomes of filamentous fungi contain numerous enzymes for degradation of starch, cellulose, lignin and similar plant polymers [17-19]. However, these predictions suffer from some limitations. As certain targeting sequences are not recognized, the predictions may contain proteins which are retained in cellular organelles. Most cell wall associated proteins can be predicted, but due to the fluctuating nature of the cell wall during growth and budding a fraction of these may be released from the cell wall structure and add to the secretome. Finally the actual composition of the secretome will depend on growth conditions and the actual expression of the genes encoding potentially secreted proteins. Therefore the extracellular proteome of *P. pastoris *was analyzed here and compared to the predicted secretome.

Substrate uptake kinetics determine growth kinetics and the characteristics of biotechnological processes. *P. pastoris *is described as a Crabtree-negative yeast, featuring respiratory metabolism under glucose surplus [20]. A major reason for the easy growth to high biomass concentrations is assumed in the endowment with hexose transporters and their features. We report here the determination and analysis of the *P. pastoris *draft genome sequence and its application in correlating *in silico *and mass spectrometric analysis of the extracellular proteome. Furthermore, a comparative analysis of hexose transporters allows drawing conclusions towards glucose uptake kinetics, a major determinant of growth and bioprocess characteristics in relation to substrate supply. Additionally, a web based database with search functions and annotation data for analysis of the genome sequence is reported.

## Results

### Sequencing

The genome of *P. pastoris *was sequenced using two next generation sequencing methods. First a Roche GS-FLX run was used to take advantage of the longer reads (400 nts) of this method, which was then complemented by a paired end run with the short read method of Illumina Genome Analyzer (36 nts) to improve the quality of the sequence. The combined result of both methods was a draft genome of 326 assembled contigs of which 93 were larger than 10 kb and 60 between 1 and 10 kb. The longest contig comprised 419,475 nts and the shortest 128 nts. 125 of the 326 contigs could be aggregated into 38 supercontigs. Overall 9,405,451 bases were sequenced with a coverage of 22× with Roche GS-FLX and 60× with Illumina GA. Key statistical data of the draft genome are presented in table 1.

**Table 1:** Genome statistics overview

**Sequencing Data:**	
Total DNA bases after Roche GS FLX	9,408,251
Average coverage Roche GS FLX	22
Total DNA bases after Illumina GA	9,405,451
Average coverage Illumina GA	60
Number of reads Roche FLX	562,515
Number of reads Illumina GA	15,761,520
Number of contigs	326
Contigs > 1 kbp	153
Largest contig	419,475
Smallest contig	128
Average contig size	28,906
GC content	41.34%
	
**Gene Prediction Data:**	
Predicted ORFs	7,935
Manually curated number of ORFs	5,450
Thereof ORFs with introns	741
Truncated ORFs	194
ORFs with annotation	4,257
GC content coding regions	41.90%
	
**RNA Prediction Data:**	
tRNA genes	149
5S rRNA	14
rRNA cluster (18S, 26S, 5.8S rRNA)	1

### Gene prediction

We initially predicted 7,935 open reading frames using two different gene finders. Manual curation reduced this number to 5,450 ORFs. The eukaryotic gene finder Augustus has been pre-trained on a number of datasets including various yeast species. Of these, *Candida guilliermondii*, *Debariomyces hansenii *and *Pichia stipitis *were selected for their relatively close relation to *P. pastoris *(based on sequence similarity), and *Saccharomyces cerevisiae *as a reference yeast species with the best sequence annotation. In addition the prokaryotic gene finder Glimmer3 was applied since many eukaryotic gene finders overpredict intron containing genes. As yeast genomes are generally compact a large amount of intron containing genes was not expected. All putative ORFs < 100 nts or comprising a starting codon other than ATG were excluded from the set except for genes on contig borders. 194 of the predicted genes are truncated because they crossed contig borders. Ribosomal RNAs were annotated by homology to *S. cerevisiae *rRNAs. Contrary to *S. cerevisiae*, the 5S rRNA is not part of the cluster containing 18S, 26S and 5.8S rRNA but spread across the genome. 149 transfer RNAs were identified using tRNA Scan, which is lower than the average number of tRNAs identified in other yeasts (216 on average).

### Functional Annotation

**Figure 1:**
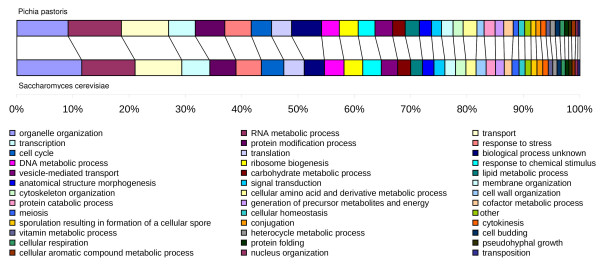
**Categorization of the *P. pastoris *annotated genome compared to *S. cerevisiae***. The GO functional groups are displayed based on their relative representation with annotated ORFs.

Functional annotation was performed computationally with a reciprocal best hit (RBH) strategy, using BLAST [21] searches against a selected dataset of the subphylum *Saccharomycotina *to which *P. pastoris *also belongs, and the Uniprot database. All *P. pastoris *genes and proteins that were publicly available at the NCBI (National Center for Biotechnology Information) were manually compared against our predictions. The native genes and proteins were present in our set. The average identity between these genes deposited in NCBI and their homologs in the present genome sequence was 95%. For all proteins that were predicted to be secreted and all others that are discussed here the functional annotation was manually curated. The distribution in GO functional terms of all functionally annotated ORFs was compared to *S. cerevisiae *(figure 1). The distribution is rather similar with differences observed mainly in the groups organelle organization, protein modification, lipid, amino acid and cofactor metabolism.

### Secretome

To validate the secretome prediction pipeline (see Materials and Methods) used for *P. pastoris*, it was applied to the *S. cerevisiae *proteome beforehand. The majority of proteins which were described to be extracellular in the Saccharomyces genome database SGD [22] were found in the secreted dataset, for the rest a GPI-anchor signal was predicted. Due to the good performance of the prediction pipeline with *S. cerevisiae *and the successful application of similar methods for *K. lactis *[15] and *C. albicans *[16] respectively, a high accuracy for the secretome predictions was expected for *P. pastoris *as well. The predicted secretome of *P. pastoris *comprises 88 putative proteins of which 55 could be functionally annotated. Additionally, 172 ORFs were predicted to encode proteins entering the general secretion pathway but being localized in different cellular compartments (for the complete list see additional file 1). Obviously the secretome prediction cannot easily discriminate between ER/Golgi localized and secreted proteins, as the chaperone Kar2 and protein disulfide isomerise (Pdi1) appear among the predictions. Therefore the experimental determination of the extracellular proteins is essential for an assessment.

**Figure 2:**
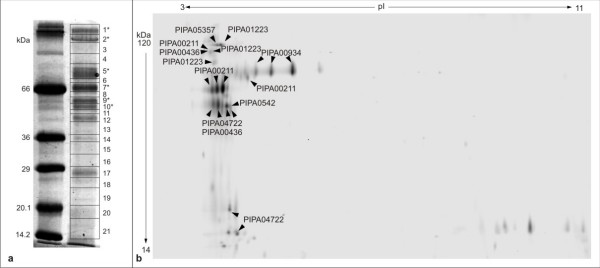
**Secretome of *P. pastoris***. (a) SDS-polyacrylamide gel. Left lane: molecular weight marker, right lane: supernatant of *P. pastoris *chemostat culture. Boxes indicate the gel slices used for LC-MS protein identification. Bands corresponding to glycoproteins are marked with an asterisk. (b) 2D electrophoresis gel of *P. pastoris *culture supernatants. Proteins identified by LC-MS are indicated.

To identify the extracellular secretome of *P. pastoris*, the strain DSMZ 70382 was grown in chemostat culture on glucose as limiting carbon source, reaching 26.4 ± 0.1 g L^-1 ^dry biomass (YDM). The supernatants contained 407 mg L^-1 ^total protein. Analysis by SDS-PAGE indicated that approximately 15 distinct protein bands, ranging from 12 kDa to 170 kDa, were present in the culture supernatant (figure 2a). On 2D gels, 28 protein spots were visible at higher abundance, at least 7 thereof being obviously isoforms of other protein spots with identical MW but different pI (figure 2b). Almost all highly abundant proteins ran at low pI values between 3 and 5.5. As the cellular viability was 99% throughout the cultivation, and total DNA content of the supernatants was 1.12 ± 0.03 μg mL^-1^, a maximum of 1% lysed cells was estimated, accounting for maximally 10% of total protein in the supernatant. Therefore, the potential contamination by intracellular protein was assumed to be minor. A 1D SDS PAGE gel was cut into 21 slices and analyzed by LC-ESI-MS/MS. Detailed data on protein identification are found in additional file 2. Twenty different proteins were identified (table 2), 12 of which appeared in more than one gel slice (additional file 2). Nine proteins ran at higher molecular weight than predicted from the sequence. Eight out of these proteins contained potential N-glycosylation sites (table 2 and additional file 2) and corresponded to detected glycoproteins (figure 2a). Apparently 6 of these proteins were subject to proteolysis. However, the proteolytic activity in the supernatants was very low (equivalent to 11 ± 0.9 ng mL^-1 ^trypsin), and in contrast to other yeast secretomes, no protein with putative proteolytic activity was identified. Fourteen of the proteins identified by homology are obviously secreted or cell wall bound, 6 of them with homology to glucanases. The other proteins with extracellular localization comprise 7 cell wall modifying enzymes and 1 secreted protein of unknown function. Four proteins are homologous to intracellular proteins (including glyceraldehyde phosphate dehydrogenase which has been described to be also located at the cell wall in *S. cerevisiae *[23], and for 2 no similarity was found. The putative intracellular proteins mainly comprise glycolytic enzymes and ribosomal proteins which are highly abundant on glucose [24]. A comparison of predicted to identified secretome reveals a good correlation of prediction, putative function, and experimentally determined localization (table 2). All proteins homologous to intracellular proteins were predicted to be intracellular, and only for 2 of the 14 putatively secreted proteins the prediction was unclear or slightly below threshold.

**Table 2:** Secreted proteins of *P. pastoris*

**PIPA ID**	**Predicted function**	**theoretical pI/MW [kDa]**	**Predicted N-glycosylation sites**	**Predicted localization**
PIPA00211	Covalently-bound cell wall protein of unknown function	5.01/45.73	1	secreted

PIPA00246	hypothetical fungal hexokinase	5.98/24.92	1	no SP

PIPA00436	Cell wall protein related to glucanases	4.83/36.07	0	secreted

PIPA00545	Cell wall protein related to glucanases	4.33/45.02	2	secreted

PIPA00748	O-glycosylated protein required for cell wall stability	4.22/31.86	1	secreted

PIPA00934	SCP-domain family protein, unknown function, extracellular	5.55/31.72	0	secreted

PIPA00956	60S ribosomal protein L18A	9.92/21.82	1	no SP

PIPA01008	GAS1; Beta-1,3-glucanosyltransferase	3.98/57.20	4	secreted

PIPA01010	GAS1; Beta-1,3-glucanosyltransferase	3.99/58.37	5	secreted

PIPA01223	potential cell wall glucanase	4.34/49.39	0	secreted

PIPA01958	Endo-beta-1,3-glucanase	4.03/33.76	1	secreted

PIPA02332	no similarity found	6.01/23.64	2	no SP

PIPA02510	Glyceraldehyde-3-phosphate dehydrogenase	6.24/35.74	1	no clear SP

PIPA02524	glucan 1,3-beta-glucosidase similar to *S. cerevisiae *EXG1 (YLR300W)	4.51/46.22	1	secreted

PIPA02544	aldehyde dehydrogenase, Adh2p [*S. cerevisiae*]	6.00/36.86	0	no SP

PIPA03955	endo-1,3-beta-glucanase [*P. stipitis *CBS 6054], Dse4p [*S. cerevisiae*]	4.70/109.45	5	secreted

PIPA04722	Cell wall protein with similarity to glucanases	5.18/32.95	0	secreted

PIPA05357	no similarity found	4.25/66.46	1	no SP, 2 TM

PIPA05673	YLR286Cp-like protein [*S. cerevisiae*], endochitinase	4.05/71.87	1	no clear SP

PIPA05771	Chitin deacetylase, Cda2p [*S. cerevisiae*]	5.25/34.66	2	secreted, lower probability

List of identified secreted proteins, with theoretical pI and theoretical MW, and information on the predicted localization (SP = signal peptide, TM = transmembrane domain).

### Hexose transporters

Fourteen putative sugar transporters all belonging to the major facilitator superfamily (MFS) were identified by sequence similarity. All *P. pastoris *sugar transporters feature the classical 12 transmembrane domains, and contain the PESP motif and at least one of the two sugar transporter signature sequences. Contrary to *S. cerevisiae*, which comprises 20 isogenes for low and high affinity hexose transport, only two putative transporters with sequence similarity to *S. cerevisiae *transporters are present in the *P. pastoris *genome. While PIPA00236 possesses more than 60% identity to *S. cerevisiae *HXT-family proteins, and the low-affinity transporters of *Kluyveromyces lactis *Rag1 [25] and *Hansenula polymorpha *Hxt1 [26] on the amino acid level, PIPA08653 shows only low similarity (max. 37% identity/58% positives) to these proteins as well as to other *P. pastoris *sugar transporters. Although all 5 conserved amino acids that have been postulated to be required for high affinity transporters in *S. cerevisiae *Hxt2 [27] are present also in the respective translated protein sequence of *P. pastoris *gene PIPA00236, disruption of the gene led to impaired growth on high concentrations of glucose (2%). Disruption of PIPA08653 did not show a distinct growth phenotype. This indicates that PIPA00236 encodes the major low affinity glucose transporter in *P. pastoris*.

**Figure 3:**
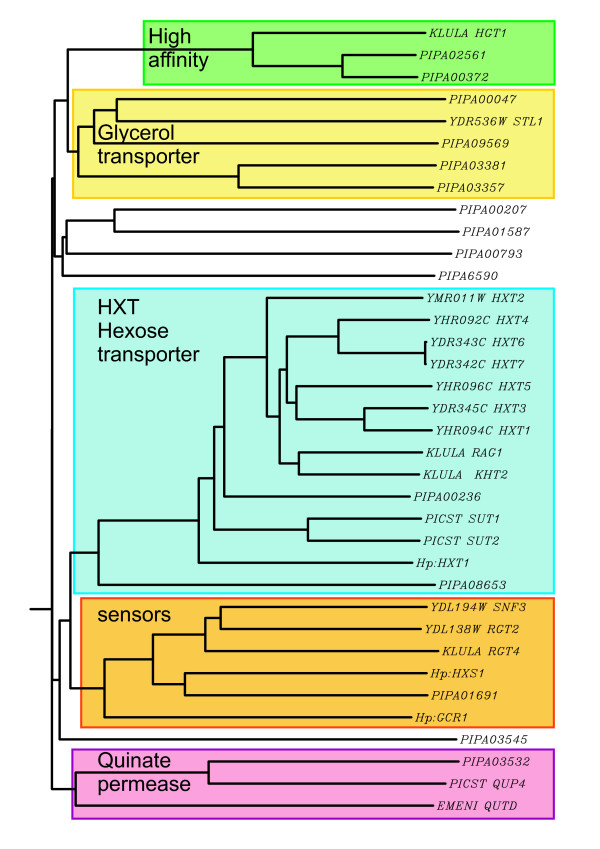
**Branch length dendrogram of sugar transporters and related proteins of different yeasts**. Putative hexose transporters and sensors and related proteins were aligned with ClustalW, and clusters of functional categories are highlighted. High affinity = high affinity glucose transporters; glycerol transporters = H^+^/glycerol symporter; HXT = low affinity *S. cerevisiae *hexose transporter family; sensors = transporter-like glucose sensors; quinate permease = homologs to fungal quinate permeases. ORF IDs relate to: PIPA = *P. pastoris*; Ynnnnnn = *S. cerevisiae*; KLULA = *K. lactis*; PICST = *P. stipitis*; Hp = *H. polymorpha*; EMENI = *Emericella nidulans*. ORFs not highlighted are homologous to other substrate transporters with sequence similarity to hexose transporters.

For high affinity transport, two *P. pastoris *proteins (PIPA02561 and PIPA00372) with high sequence similarity (>65% identity) to *K. lactis *high affinity glucose transporter Hgt1 were identified (see figure 3). The potential transporter-like hexose sensor is encoded by PIPA01691, and lacks the C-terminal "glucose sensor domain" as do the respective orthologous sensors in *H. polymorpha *(Hxt1) and *Candida albicans *[26]. Additionally a gene with similarity to quinate permease of *P. stipitis *and filamentous fungi was identified, which has putative orthologs in many other yeast species, but is missing in *S. cerevisiae*. According to Barnett et al. [28]* P. pastoris *cannot utilize quinate as a carbon source, although some of the genes required for the utilization of quinate are part of the shikimate pathway leading to the production of aromatic amino acids, and are present as part of the pentafunctional AROM protein. However, regulatory proteins of the quinate pathway are missing in the genome of *P. pastoris*. Interestingly, *P. pastoris *possesses four transporters that are highly similar to putative glycerol transporters from *K. lactis *(KLLA0A03223g) and *Yarrowia lipolytica *(YALI0F06776g), and weakly similar to the *S. cerevisiae *glycerol transporter Slt1. Sequence similarities of the proteins discussed above to their respective orthologs in *S. cerevisiae*, *P. stipitis*, *H. polymorpha*, *K. lactis*, and *Emericella nidulans *are illustrated in figure 3.

### Database, genome browser

To make the genomic data accessible it was loaded into a relational database. For visualization a genome browser was installed on a web server and connected to the database.

The genome browser of *P. pastoris *is publicly available at  [29].

The draft genome sequence data are deposited at EMBL- EBI, accession number CABH01000001 – CABH01000326.

## Discussion

The predicted size of the haploid genome of *P. pastoris *[30] was confirmed here to comprise 9.4 Mb, which is smaller than the genomes of other yeasts, spanning from 10–20 Mb [31]. Nevertheless the number of functionally annotated genes is comparable to other yeasts, which can be attributed to the fact that *P. pastoris *contains fewer genome redundancies compared e.g. to S. *cerevisiae *and *D. hansenii*, which have undergone genome duplications followed by partial genome losses during evolution [32]. While *P. pastoris *contains specific subclasses of genes for methanol metabolism and peroxisome synthesis, structure and degradation which are present only in methylotrophic yeasts, most metabolic enzymes are present only in single copies, and the number of secreted proteins is low. To verify the quality of gene prediction, all 173 *P. pastoris *genes and 245 proteins currently deposited in NCBI were BLAST searched among the predicted gene list. All of the *P. pastoris *specific genes were present, indicating a high quality of gene prediction.

The secretomes of *K. lactis *and *C. albicans *have been predicted computationally [15,16], yielding 178 ORFs of *K. lactis *and 283 of *C. albicans*. The *C. albicans *secretome apparently is more complex and contains numerous lipases, proteases and agglutinin-like proteins, while both for *K. lactis *and *P. pastoris *only few enzymes apart from glucanases and chitin modifying enzymes appear. As *P. pastoris *utilizes only few carbon sources [28] it appears obvious that neither proteolytic, lipolytic or saccharolytic activities are secreted for substrate utilization. Yeast glucanases and chitinases are required for cell wall plasticity during cell growth and division [33]. While these enzymes are commonly regarded to be cell wall associated, it is plausible that they reach the culture supernatant during cell wall remodelling, indicating that a distinct border cannot be drawn between cell wall and the exterior space.

**Figure 4:**
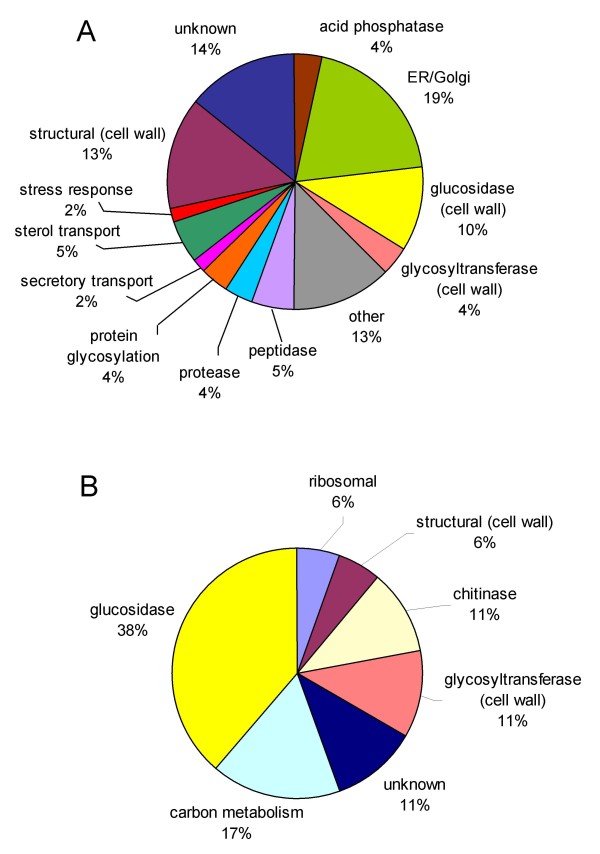
**Categorization of *P*. *pastoris *secretome**. (a) predicted and (b) detected secretome based on GO terms. Proteins without *S. cerevisiae *homologs are classified as "unknown".

Fourteen of the 20 proteins identified in the culture supernatant of *P. pastoris *were homologous to proteins implicated in cell wall or extracellular functions. No other secretory enzyme homologs were identified, further indicating that cell wall associated proteins are the essential constitutents of the secretome of glucose grown *P. pastoris*. The computationally predicted secretome contains all secreted proteins plus mainly soluble cellular proteins containing a signal peptide but no transmembrane domains. Thus these predictions obviously overestimate secretory proteomes (figure 4). The culture supernatant of *K. lactis *contained significantly more (82) proteins [15] of which 34 were predicted to be secreted or cell wall bound, and the rest were assumed to be localized either to the ER or the cytosol. The latter group of proteins indicates a significant release of intracellular proteins in this study, probably by cell lysis due to the culture conditions.

The low concentration, together with the small number of actually secreted proteins from *P. pastoris *highlights a major advantage of this protein production system, as secreted products are much less contaminated with host cell protein. Jahic et al. [34] have shown that host cell protein released from *P. pastoris *grown on methanol mainly derives from cell lysis, which occurs to a much lower extent upon growth on glucose. Combined with the fact that strong promoters for use on glucose are available [34,35], these data provide convincing arguments for a reconsideration of methanol based protein production with *P. pastoris*. The toxicity of methanol and several of its metabolites is the main reason for cell lysis and consequently also protease leakage to the culture supernatant. Additionally other host cell proteins are released, leading to significant contamination of protein products. A common approach to reduce product proteolysis is the knock out of protease genes. However, multiple protease knockout strains tend to be growth retarded, so that it appears reasonable to employ a production strategy based on glucose media which avoids the detrimental effects of methanol at all. Detailed knowledge of the secreted host cell proteins, as presented here, can have a strong positive effect on product purification and quality control, as specific assays can be developed. Additionally a knock out of major secreted proteins can reduce the host cell protein load significantly [36].

Substrate uptake kinetics determines growth kinetics and the characteristics of biotechnological processes. The fermentative (Crabtree-positive) yeast *S. cerevisiae *consumes glucose at high rates when supplied with high concentrations. This exceptionally high glucose uptake rate is attributed to high abundance of hexose transporters, encoded by more than 10 isogenes [37]. Respiratory (Crabtree-negative) yeasts limit glucose uptake, as they contain few hexose transporter genes, encoding energy dependent symporters with high affinity to glucose [38]. The endowment of *P. pastoris *with hexose transporters is in good accordance to other respiratory yeasts such as *K. lactis*, *H. polymorpha *and *P. stipitis*, all having a reduced number of hexose transporters in comparison to *S. cerevisiae*. Moreover, Crabtree-negative yeasts usually exhibit K_m _values in the micromolar range for glucose [37], due to their very high-affinity transporters such as *K. lactis *Hgt1, which is an ortholog of *P. pastoris *PIPA02561 and PIPA00372. While K_m _values for *P. pastoris *specific transporters remain to be determined in future, conclusions to glucose uptake behavior can be drawn. Accordingly, specific glucose uptake rate is limited to q_Smax _= 0.35 g g^-1 ^YDM h^-1 ^(at growth rates near μ_max _= 0.193 h^-1^) in *P. pastoris *chemostat cultivations [39], in comparison to q_Smax _= 2.88 g g^-1 ^YDM h^-1 ^in fully aerobic *S. cerevisiae *[40]. The limited glucose uptake prevents Crabtree-negative yeasts such as *P. pastoris *from extensive overflow metabolism, which leads to the aerobic formation of ethanol and a reduced biomass yield at high external glucose concentrations in *S. cerevisiae*. This difference is also reflected in the very high biomass concentrations (more than 100 g l^-1^) that can be achieved in *P. pastoris *cultivations. For heterologous protein production, aerobic ethanol formation is a substantial problem, because it lowers the yield of the desired product due to a lower biomass concentration.

Interestingly, *P. pastoris *contains four genes encoding putative H^+^/glycerol symporters, contrary to all other sequenced yeasts up-to-date. Consequently, the maximum glycerol uptake rate of *P. pastoris *is q_Glycerol_max _= 0.37 g g^-1 ^YDM h^-1^. This is substantially higher than the uptake rates reported for *S. cerevisiae *(q_Glycerol_max _= 0.046 g g^-1 ^YDM h^-1^) and many other yeast species [41]. The ability to grow on glycerol as a single carbon and energy source – a mode of cultivation widely applied for generation of biomass with *P. pastoris *prior to methanol induction or glucose fed batch – is dependent on the activity of a constitutive salt-independent active glycerol transport by the H^+^/glycerol symport and has also been reported for *Pichia sorbitophila *and *Pichia jadinii *[41]. Specific growth rates of these yeasts on glycerol are similar to the specific growth rates that can be obtained on glucose (e.g. for *P. pastoris *on mineral media μ_Glycerol_max _= 0.26 h^-1^, μ_Glucose_max _= 0.19 h^-1^), whereas yeasts lacking the activity of such a type of carrier have significantly reduced growth rates on glycerol. The high specific glycerol uptake rate, enabled by the exceptional endowment with specific transporters emphasizes the suitability of glycerol as a substrate for biomass growth.

## Conclusion

The availability of genome data has become an essential tool for cell and metabolic engineering of biotechnological production organisms. This work highlights major advantages of *P. pastoris *as a protein production platform and the benefits of glycerol/glucose based production technology. Apart from lower heat production and oxygen demand compared to methanol based processes, glucose grown cultures display higher viability and essentially no protease release to the culture supernatant. Furthermore detailed insights into the sugar transport will enable rational modulation of substrate fluxes, especially for efficient metabolite production.

## Material and methods

### Strain

The *P. pastoris *type strain (DSMZ 70382 = CBS704) was selected as the source of genomic DNA, and used for all experimental work. Genomic DNA was prepared as described in Hohenblum et al. using the Qiagen Genomic G-20 kit [42].

### Sequencing

Genomic DNA was sequenced by GATC Biotech AG, Konstanz (Germany) with a Roche GS FLX-Titanium Series complemented by an Illumina Genome Analyzer paired end run. The reads were assembled with SeqMan NGen by DNASTAR. To verify the sequencing quality all *P. pastoris *gene and protein sequences available at NCBI were downloaded and the sequences were compared using BLAST searches.

### Gene prediction and annotation

Gene prediction was performed with the eukaryotic gene finder Augustus [43] using the option for overlapping genes as well as the prokaryotic gene finder Glimmer3 [44]. Predicted open reading frames were kept if they were longer than 100 nucleotides and started with ATG, except for genes predicted on contig boarders. The ORF sets were merged and made non redundant using the clustering program cd-hit-est [45] with a similarity cut-off of 95%.

Annotation was done by a reciprocal protein BLAST against a dataset consisting of the publicly available *Saccharomycotina *species and the UNIPROT protein database with an E-value threshold of 10^-10^. All *P. pastoris *proteins and genes available at NCBI, all proteins that were predicted to be secreted and all sugar transporters were manually curated. Gene Ontology annotation was done for all proteins with a homolog in *S. cerevisiae*.

Ribosomal RNA annotation was done through homology with *S. cerevisiae *using nucleotide BLAST against the *P. pastoris *contigs, and the results were manually analyzed. tRNAs were localized using the program tRNAscan-SE [46]. Gene predictions were manually curated using BLASTx.

### *In silico *secretome prediction

A similar method was used as described to predict the secretomes of *K. lactis *[15] and *C. albicans *[16], respectively. The prediction pipeline included SignalP 3.0 [47,48] to identify the N-terminal signal peptide, Phobius [49] to predict the transmembrane topology, GPI-SOM [50] and the fungal version of big-PI [51] for GPI anchor prediction, TargetP [52] to exclude all proteins with predicted mitochondrial localization. Additionally WoLF PSORT [53] was used for general localization prediction.

Proteins were considered to be secreted when an N-terminal signal peptide existed but neither a transmembrane domain (except one within the first 40 residues), nor a GPI-anchor, nor any localization signal to other organelles were identified.

The prediction pipeline was tested on an *S. cerevisiae *dataset of 5,884 proteins which was downloaded from the Saccharomyces Genome Database SGD [22].

### Experimental secretome analysis

*P. pastoris *DSMZ 70382 was grown in fully aerobic chemostat cultures on minimal medium with glucose as carbon source until steady state (biomass yield and RQ constant for at least 2 residence times). Detailed data on media compositions, fermentation data and the analysis of culture supernatant can be found in additional file 3. Culture supernatants were concentrated by acetone precipitation and subjected to 1D SDS-PAGE on a 12% PAA gel and 2D-DIGE, respectively. For 2D-DIGE supernatant protein was Cy5 labelled and separated on a IPGDryStrip (3-11NL) in the first dimension, followed by SDS-PAGE on a 12% PAA gel as described in Dragosits et al. [24]. 1D gel lanes were cut into 21 slices, and protein spots from CBB stained 2D gels were picked. After tryptic digest, samples were analyzed by reversed-phase chromatography (UltiMate 3000 Capillary LC-system, Dionex) coupled with ESI MS/MS analysis (Q-TOF Ultima Global, Waters). The obtained mass spectra were subsequently analysed using X!Tandem 2008.12.01 [54]. The identified proteins had to meet the following criteria: protein score e-value ≤ 10^-5 ^with at least 2 peptides per protein. Glycoproteins were detected by SDS-PAGE and blotting of proteins onto a nitrocellulose membrane followed by detection via Concanavalin A and Horseradish peroxidase. Putative N-glycosylation sites were identified with NetNGlyc 1.0 server [55].

### Analysis of hexose transporters

*P. pastoris *ORFs encoding putative sugar transporters were identified by sequence similarity using BLAST. Multiple sequence alignment of the respective protein sequences to previously identified hexose transporters and sensors from other yeasts was performed by ClustalW [56] using BLOSUM weight matrix, and a dendrogram with branch length was generated. Additionally an integrated search in PROSITE [57], Pfam, PRINTS and other family and domain databases was performed with InterProScan [58] for all these protein sequences.

Disruption cassettes for PIPA00236 and PIPA08653 were generated by PCR (primers: PIPA08653FW: ATGGCAGGTATTAAAGTTGGATC; PIPA08653BW: TACTGCCATCTGCTTCTTTC; PIPA00236FW: GCAGGAGAATAGTCCAGTTTAC; PIPA00236BW: TTCATAGCCTCGTCGACTCTG). 200–300 bp each up- and downstream of the start codon were exchanged for the Zeocin resistance cassette. These cassettes were introduced into the genome of *P. pastoris *DSMZ 70382 by electroporation, and clones were selected on YP plates containing 1% yeast extract, 2% peptone, 2% agar-agar, 2% glycerol and 25 μg mL^-1 ^Zeocin. Positively growing clones were then analyzed for their growth behavior on YP plates containing either 2% glycerol, 2% glucose or 0.01% glucose for 48 h at 28°C.

### Genome Database

The gene predictions were parsed into GFF file format and loaded into a Chado [59] database which is designed especially to hold a wide variety of biological data.

Gbrowse [60], the Generic Genome Browser, was installed on a web server in the latest stable version (1.69) and configured to display the genomic data from the Chado database.

## Competing interests

The authors declare that they have no competing interests.

## Authors' contributions

DM initiated and coordinated the *P. pastoris *genome project. AG and AR were responsible for genome annotation and analysis. AG predicted the secreted proteins. MD performed the chemostat cultivations and 2D-gel electrophoresis. AR developed the genome database. JS performed the MS identification of the secreted proteins. FA coordinated and supervised proteomics. MM, MK and MS contributed to annotation. BG carried out the analysis of the hexose transporters and contributed to gene annotation. DM, AG, MD, MM and BG wrote the final text of the manuscript.

## Supplementary Material

Additional file 1 **Predicted secretome of *P. pastoris***. Predicted localization of all genes containing a predicted signal peptide. The output of the prediction pipeline is given, as well as ORF and gene names and descriptions of *S. cerevisiae *homologs, if available. Click here for file

Additional file 2 **Summary of identified proteins**. List of mass spectrometry identified proteins on both 1D and 2D gels, including protein scores and all individual peptides with corresponding peptide scores. Click here for file

Additional file 3 **Chemostat cultivation data**. Detailed chemostat cultivation data including culture medium composition and evaluation of DNA, RNA and protein content of the supernatant. Click here for file
